# Surgery for non-Meckel’s small-bowel diverticular perforation: two case reports and a literature review

**DOI:** 10.1186/s40792-024-02000-x

**Published:** 2024-10-08

**Authors:** Naoki Matsuya, Akifumi Kuwabara, Nobuhiro Morioka, Tadashi Tanabe, Nobuyuki Musha, Ken Nishikura, Toshihiro Tsubono

**Affiliations:** 1 Department of Surgery, Saiseikai Niigata Hospital, 280-7, Teraji, Niigata, Niigata 950-1104 Japan; 2 Department of Pathology, Saiseikai Niigata Hospital, 280-7, Teraji, Niigata, Niigata 950-1104 Japan

**Keywords:** Multiple small-intestinal diverticula, Emergency surgery, Small intestinal perforation

## Abstract

**Background:**

Similar to colonic diverticula, small-intestinal diverticula are often asymptomatic, but may cause life-threatening acute complications. Non-Meckel’s small-bowel diverticular perforation is rare, and the rate of mortality is high. However, there is currently no consensus regarding its therapeutic management.

**Case presentation:**

Case 1: A 73-year-old Japanese man with localized lower abdominal pain was referred to our hospital. Enhanced computed tomography (CT) revealed diverticulitis of the small intestine, which was managed conservatively. Four days after admission, abdominal pain worsened, and repeat CT revealed extraintestinal gas. Emergency surgery was performed for the segmental resection of the perforated jejunum with anastomosis. Case 2: A 73-year-old Japanese woman was transferred to our hospital with small-bowel perforation. CT revealed scattered diverticula in the small intestine and extraintestinal gas around the small-intestinal diverticula. Emergency surgery was performed for the segmental resection of the perforated jejunum with anastomosis.

**Conclusions:**

Conservative treatment for small-bowel diverticular perforation may be attempted in mild cases; however, surgical intervention should not be delayed. Segmental resection of the affected intestinal tract with an anastomosis is the standard treatment. Residual diverticula should be documented because of the possibility of diverticulosis recurrence.

**Supplementary Information:**

The online version contains supplementary material available at 10.1186/s40792-024-02000-x.

## Background

A diverticulum can be located at any location in the gastrointestinal tract, from the upper esophagus to the colon. Non-Meckel small-bowel diverticula are rare, with an incidence of less than 1% in the general population [1]. Although most patients with small-bowel diverticula are asymptomatic, some present with chronic symptoms, such as diarrhea, malabsorption, chronic abdominal pain, and discomfort. It may also cause fatal acute complications, such as intestinal hemorrhage, bowel obstruction, and perforation [2, 3].

Non-Meckel’s small-bowel diverticular perforation is uncommon, and there is no consensus on its therapeutic management. Here, we report two cases of emergency surgery for multiple non-Meckel small-bowel diverticular perforations and discuss their management.

## Case presentation

### Case 1

A 73-year-old Japanese man was referred to our hospital with localized lower abdominal pain associated with vomiting after the consumption of raw fish. His medical history revealed that he had undergone a sigmoidectomy for sigmoid colon cancer. The patient had no history of allergies and was not on any medication. Physical examination revealed abdominal tenderness and localized guarding of the right upper quadrant. The vital signs were stable except for a fever of 38 °C. Laboratory tests revealed a raised white blood cell count of 10,200/μL and C-reactive protein level of 0.60 mg/dL.

**Fig. 1:**
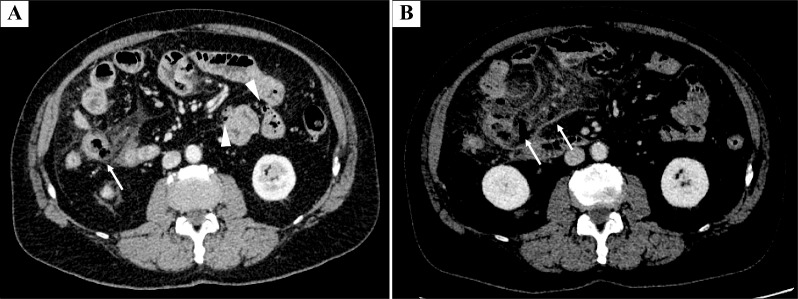
Selected computed tomography (CT) images show the clinical course of perforated small-bowel diverticulum. **A** Enhanced CT scan at the time of initial presentation showing an inflammatory change in the mesentery adjacent to the thickened small bowel (white arrow). A scattered diverticulum in the small intestine is also noted (arrowheads). **B** Repeat enhanced CT scans 4 days later showing worsening mesenteric inflammatory changes and the appearance of extraintestinal air (white arrow). These findings were suggestive of perforated diverticulitis

Enhanced computed tomography (CT) revealed a thickened partial small-bowel wall, stranding of peri-intestinal fat, and no obvious extraintestinal gas. Scattered diverticula were observed in the small intestine (Fig. 1A).

**Fig. 2:**
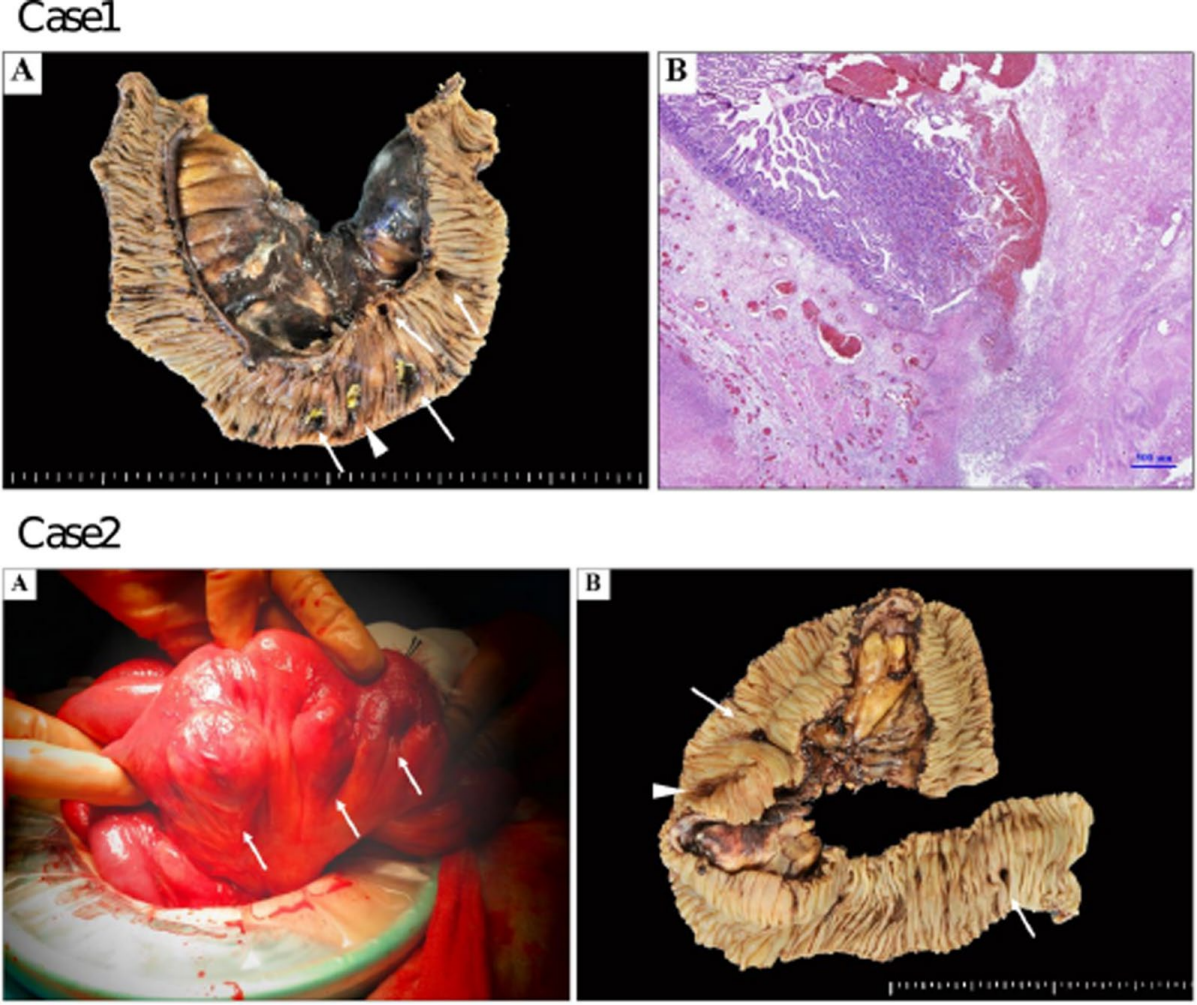
Case 1 A: Surgical specimen shows diverticula in jejunum (white arrows), one of which was perforated (arrowhead). B: Hematoxylin and eosin staining showing perforation of the pseudodiverticulum, phlegmonous peritonitis, and abscesses with many bacterial aggregations. Case 2 A: Intraoperative image of dilated loops of jejunum with multiple small-bowel diverticula (white arrows). B: Surgical specimen showing a diverticulum in the jejunum (white arrows), one of which is perforated (arrowhead)

The patient was admitted and managed conservatively with a diagnosis of small-bowel diverticulitis. Four days after admission, the patient complained of abdominal pain and blood tests revealed a markedly elevated inflammatory response. Repeat CT revealed extraintestinal gas around the small-intestinal diverticula (Fig. 1B). Emergency surgery was performed for the segmental resection of the perforated jejunum, followed by functional end-to-end anastomosis. The final pathological analysis revealed perforation of the pseudodiverticulum, phlegmonous peritonitis, and an abscess with numerous bacterial aggregations (Fig. 2). The patient recovered well after surgery with antibiotic therapy and was discharged on postoperative day 11.

### Case 2

A 73-year-old Japanese woman was transferred to our hospital with small-bowel perforation. She developed abdominal pain after ingesting raw fish and was observed fasting for 2 days, her symptoms did not improve, and she visited a nearby hospital. Her medical history revealed that she had undergone appendectomy for appendicitis and had untreated diabetes. The patient was on medication for psychosomatic illnesses and had no history of allergies. Physical examination revealed widespread abdominal tenderness and guarding, primarily in the left abdomen. The vital signs were stable except for a fever of 37.9 °C. Laboratory tests revealed a raised white blood cell count of 9000/μL and a high C-reactive protein level of 22.7 mg/dL and a high HbA1c level of 9.6%.

**Fig. 3:**
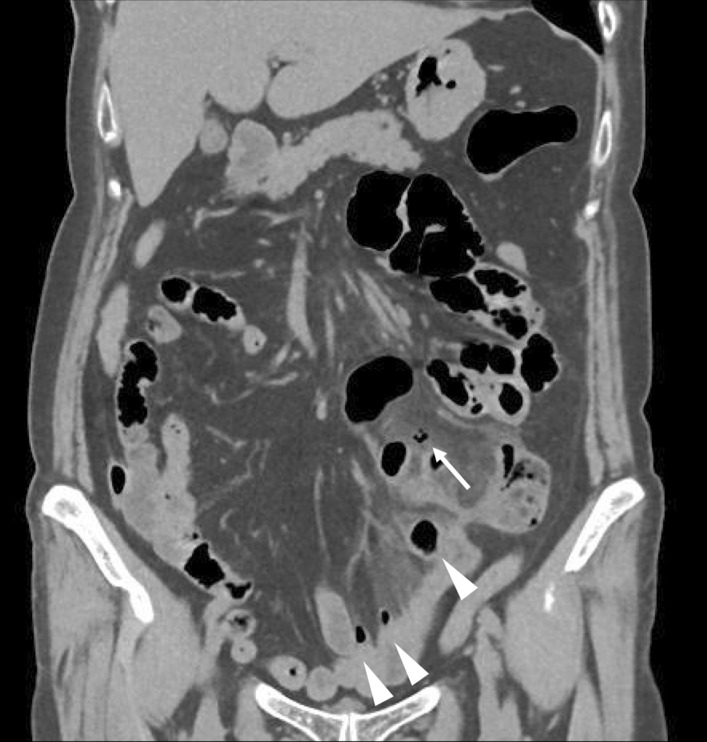
Selected CT images showing multiple diverticula in the small intestine (arrowheads), mesentery inflammatory changes, and extraintestinal air (white arrow)

**Fig. 4:**
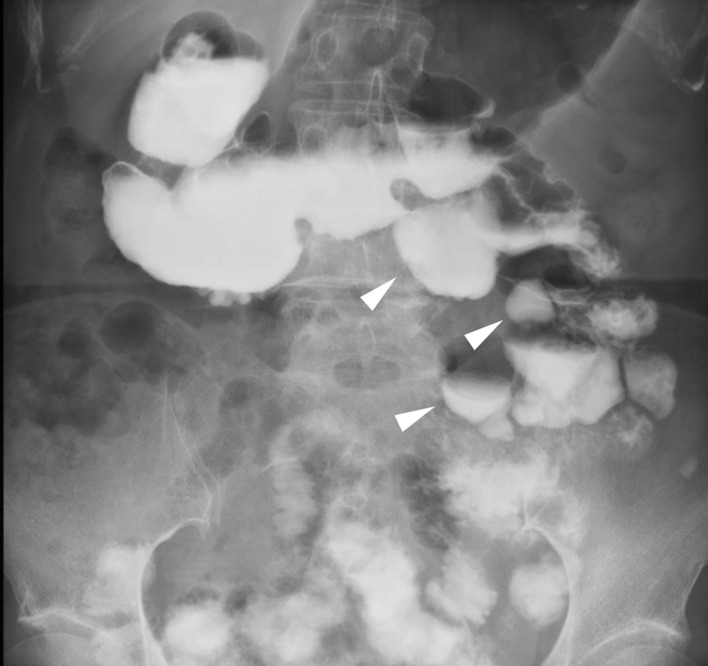
Small-bowel series performed at 2 months postoperatively showing multiple and dilated jejunal diverticula (arrowhead)

CT revealed scattered diverticula from the small intestine to the colon, a thickened partial small-bowel wall, and extraintestinal gas around the small intestine diverticula (Fig. 3). Emergency surgery was performed for the segmental resection of the perforated jejunum, followed by functional end-to-end anastomosis. Intraoperative findings and surgical specimens showed diverticula in the jejunum, one of which was perforated. The final pathological analysis revealed a perforation of the pseudodiverticulum and secondary serositis (Fig. 2). The patient recovered well after surgery with antibiotic therapy and was discharged on postoperative day 7. Later, a small-bowel series revealed numerous small-bowel diverticula (Fig. 4).

## Discussion

Non-Meckel small-bowel diverticula are rare, and the frequency of autopsy cases was reported by Edwards as 9 out of 2820 cases (0.31%). According to Edward’s *locus minoris resistentiae* theory, increased intestinal pressure causes a hernia-like prolapse of the mucosa and submucosal tissue out of the intestinal wall at the mesenteric vascular penetration zone, where the muscular layer is underdeveloped, often resulting in multiple pseudodiverticula in the mesenteric attachment zone [1]. Perforation occurs mainly in the mesenteric leaves of the small intestine, resulting in a mesenteric abscess. In our case, diverticula and perforations were also observed on the mesenteric border. We summarized the differences between Meckel’s diverticulum and non-Meckel's small-bowel diverticulum (Table 1).

**Table 1:** Comparison of Meckel’s diverticulum and nonMeckel’s small-bowel diverticulum

	Meckel’s diverticulum	Non-Meckel’s small-bowel diverticulum
Etiology	Congenital	Acquired
Prevalence	2%	Less than 1%
Common age of onset	Before 2 years old	After 60 years old
Location	Two feet from the ileocecal valve	Throughout the small intestine
	Anti-mesenteric border	Along the mesenteric border
Number of diverticulum	Single	Single to multiple
Types of diverticulum	True diverticulum	Pseudodiverticulum
Heterotopic mucosa	Presence	Absence
Schematic drawing		

As with colonic diverticula, small-intestinal diverticula are often asymptomatic, but they may cause life-threatening acute complications, such as bleeding, volvulus, obstruction, diverticulitis, and perforation, which can lead to major diagnostic and therapeutic problems [4]. In particular, the mortality rate of diverticular perforation is high, ranging from 21 to 40%, and is closely related to delays in diagnosis and older age [5, 6].

Owing to their rare incidence, there is no clear treatment strategy for small-bowel diverticular perforations. Because of the difference in the pathogenic bacteria, we assume that the same treatment protocols as for upper gastrointestinal perforation cannot be applied. Therefore, we collected cases of small-intestinal diverticular perforation since 2000–2023 and summarized recent trends in treatment strategies (Table 2) [7–32].

**Table 2:** Summary of previous reports and our cases since 2000 to 2023

Year authors	Period from onset	Age/sex	Location of lesion (number of diverticulum)	Peritoneal irritation sign	Key method of diagnosis and findings	Management	Clinical course
2003 Nightingale	4 h	83/F	Jejunum (multiple)	Yes	CT; multiple small-bowel diverticula and free gas adjacent to the mesentery	Segmental resection	Discharged POD 10
2008 Staszewicz	NA	88/M	Jejunum (multiple)	NA	CT; multiple small-bowel giant diverticula surrounded by inflammatory mesenteric fat	Segmental resection	Discharged POD 12
2009 Borgaonkar	3 days	65/M	Jejunum (1)	Yes	Sonography; multiple small-bowel loops with free fluid	Exploratory laparotomy Segmental resection	Discharged POD 10
2009 Colvin	7 days	87/M	Jejunum (NA)	Yes	CT; soft tissue stranding with multiple small locules of gas which is surrounded by small-bowel loops	Conservative	Discharged after 4 days of admission
2010 Sakpal	3 days	25/F	Jejunum (1)	Yes	CT; a thickened jejunal wall and an air-fluid-containing structure	Exploratory laparotomy Segmental resection	Discharged POD 7
2010 Butler	1 day	82/F	Jejunum (multiple)	Yes	CT; multiple small-bowel diverticula with surrounding pockets of free air	Primary closure of two sites of perforated diverticula	recovered
2013 Akbari	2 days	74/M	Jejunum (multiple)	Yes	no	Exploratory laparotomy Segmental resection	Discharged POD 7
2014 Kavanagh	2 days	63/M	Jejunum (multiple)	Yes	CT; extraluminal gas	Exploratory laparotomy Segmental resection	Discharged POD 3
2014 Baksi	1-2 days	55/M	Jejunum (1)	Yes	X-ray; dilated loops of small bowel and free gas under the diaphragm	Exploratory laparotomy Segmental resection	Discharged POD 10
2014 Levack	3 days	77/F	NA	No	CT; a small collection adjacent to the thickened small bowel	Conservative	Discharged after 5 days of admission
2015 Natarajan	7 days	58/M	Jejunum (multiple)	NA	X-ray; free air under the diaphragm C; multiple diverticula in the small intestine and air under the diaphragm	Segmental resection	Discharged POD 10
2016 Sehgal	2 days	82/M	Jejunum (multiple)	Yes	CT; a hollow viscus perforation with free air and intrapelvic inflammatory change in the mesentery	Exploratory laparotomy Segmental resection	Discharged POD 7
2017 Ejaz	on the day	87/M	Jejunum (multiple)	NA	CT; mesenteric fat stranding and a small pocket of extraluminal gas adjacent to a jejunal diverticulum	Conservative	Discharged after 5 days of admission
2017 Karas	1 day	69/M	Terminal ileum(multiple including jejunum)	No	CT; a small foci of extraluminal gas surrounding the terminal ileum	Initially conservative but worsened in the next 24 h Exploratory laparoscopy Open segmental resection	Unremarkable
2018 Syllaios	2 days	75/M	Jejunum (multiple)	Yes	CT; a small amount of extraluminal air adjacent to the jejunum	Segmental resection	Discharged POD 6
2018 Kagolanu	2 days	91/M	Jejunum (multiple)	Yes	CT; small-bowel diverticula with inflammation and a contained micro-perforation	Conservative	Treated within 2 days
2018 Alves	1 day	74/F	Jejunum (multiple)	Yes	X-ray; free gas under the right hemidiaphragm and distension of the small bowel	Exploratory laparotomy Segmental resection	Discharged POD 22
2019 Jambulingam	on the day	63/F	Jejunum (2)	No	CT; inflammatory infiltrate surrounding large jejunal diverticulum which was localized to the surrounding mesentery	Conservative	Discharged after 2 days of admission
2020 Kunishi	on the day	40/F	Jejunum (multiple)	No	CT; localized extraluminal air and panniculitis adjacent to the jejunum diverticula	Conservative	Discharged after 6 days of admission
2022 Leigh	2 days	59/F	Jejunum (multiple)	No	CT; a jejunal loop with a large diverticulum on the mesenteric side with perforation	Segmental resection	Discharged POD 6
2021 Ben	2 days	52/M	Jejunum (1)	Yes	CT; a jejunal diverticulum with surrounding inflammatory changes in the mesenteric fat	Initially conservative but worsened in the next 72 h Exploratory laparoscopy Segmental resection	NA
2021 Rajaguru	5 days	74/M	Ileum (1)	Yes	CT; inflammatory changes in the right iliac fossa with the presence of extraluminal gas locules	Exploratory laparoscopy Laparoscopic assisted right hemicolectomy	Discharged POD 4
2022 Ponce	7 days	83/M	Jejunum (multiple)	Yes	CT; air-fluid distention of the entire small bowel and small mesenteric collection	Exploratory laparotomy Segmental resection and ostomy	Died 6 h later
2022 Mejri	1 day	60/F	Jejunum (multiple)	Yes	NA	Segmental resection	Discharged POD 6
2023 Jawed	3-4 days	75/M	Ileum (3)	Yes	X-ray; dilated bowel loops and free air under the diaphragm	Exploratory laparotomy Segmental resection and a double barrel ileostomy	Discharged POD 4
2023 Dar	2 days	38/F	Jejunum (12)	Yes	X-ray; free air under the diaphragm	Exploratory laparotomy Segmental resection	Discharged POD 10
2023 Matsuya	on the day	73/M	Jejunum (multiple)	Yes	CT; a thickened partial small-bowel wall, stranding of peri-intestinal fat Repeat CT: revealed extraintestinal gas around the small-intestinal diverticula	Initially conservative but worsened in the next 96 h Segmental resection	Discharged POD 11
2023 Matsuya	2 days	73/F	Jejunum (multiple)	Yes	CT; scattered diverticula in the small intestine to the colon, thickened partial small-bowel wall, and extraintestinal gas around the diverticula	Segmental resection	Discharged POD 7

The clinical findings vary widely and patients visit the hospital with various concerns. Abdominal pain varied according to its location, severity, and progression. CT plays a major role in the diagnosis of diverticulitis in the small intestine. Localized and asymmetric thickening of the small-intestinal wall and inflammation or abscess of the periportal adipose tissue are the diagnostic criteria for small-intestinal diverticulitis, and when added to these findings of extraintestinal gas, a diagnosis of perforation or permeation can be made [33]. Non-Meckel’s small-bowel diverticula are often multiple, and the presence of diverticula in the normal small intestine may also help in the diagnosis of small-intestinal diverticulitis. In Table 2, however, there are scattered cases in which an exploratory laparotomy is selected even when CT is taken. One reason may be that small-bowel diverticular perforation is a rare disease not mentioned in the differential. The non-surgical management of perforated small-intestinal diverticula is a relatively new concept. When perforation of a small-intestinal diverticulum causes localized peritonitis and the patient remains stable, non-surgical management, such as antibiotics, bowel repose, and CT-guided aspiration of localized intraperitoneal collections, may avoid the need for surgery [18, 21, 23, 24, 31, 34]. On the other hand, emergency surgery is performed when there is remote air from the inflamed diverticulum. Patients successfully treated conservatively are often discharged from the hospital relatively early (2–6 days) [18, 21, 23, 24, 31]. Considering the high mortality rate associated with diverticular perforations of the small intestine, conservative treatment should be provided in limited cases. Even when conservative treatment is selected, surgery should be considered immediately in patients who do not improve after a few days of conservative treatment. In the early stages of the perforation, as in Case 1, it is impossible to determine whether the inflammation stays in the mesentery or spreads. A repeat CT scan may confirm the spread of extraintestinal gas and worsening of inflammatory findings.

We have summarized the main surgical techniques for non-Meckel’s small-bowel diverticular perforation (Table 3). Segmental intestinal resection with primary anastomosis is the most common procedure for perforating diverticula in the small intestine. Other surgical techniques, such as simple closure, invagination, and excision of the perforated diverticulum, should be abandoned because of their high mortality rate [35]. When the diverticula extend over the long intestinal tract, resection should be limited to the perforated or inflamed portion to avoid short-bowel syndrome. The presence of a retained diverticulum should be recorded for future reference in light of case reports of recurrent small-intestinal diverticular perforation after surgery [31, 36].

**Table 3:** Major surgical techniques for diverticular perforation of small intestine and number of cases in review

Surgical technique	Number of cases
Segmental resection (with primary anastomosis/ostomy)	19/2
Diverticulectomy	0
Simple closure of the diverticulum	1
Invagination of the diverticulum	0

## Conclusions

Perforation of non-Meckel small-bowel diverticula is rare, but the mortality rate is high and should be considered in the differential diagnosis of acute abdominal pain. Although conservative treatment has been reported, surgery should be performed promptly when symptoms worsen. Segmental resection and anastomosis of the affected intestinal tract are the standard treatments. Residual diverticula should be documented because of the possibility of diverticulosis recurrence.

## Supplementary Information

Supplementary Material 1.

## Data Availability

The data for the patients are available upon request.

## Abbreviations

CT: Computed tomography
POD: Postoperative day
