# Supplementary material for: Surgery for non-Meckel’s small-bowel diverticular perforation: two case reports and a literature review
Source: Surg Case Rep. 2024 Oct 8;10:232. doi: 10.1186/s40792-024-02000-x (PMC11461421; doi:10.1186/s40792-024-02000-x)
Supplement: Supplementary file 1 — Supplementary Material 1. [file 40792_2024_2000_MOESM1_ESM.docx]

Table 4　Details of previous reports and our cases since 2000 to 2023

| Year  authors | Chief complaint | Period from onset | Age/Sex | Location of lesion (number of diverticulum) | Peritoneal irritation sign | Key method of diagnosis and findings | Management | Clinical course |
| --- | --- | --- | --- | --- | --- | --- | --- | --- |
| 2003  Nightingale | Suddengeneralized abdominal pain | 4 hours | 83/F | Jejunum (multiple) | Yes | CT; multiple small bowel diverticula and free gas adjacent to the mesentery | Segmental resection of the affected jejunum with primary anastomosis | Discharged POD 10 |
| 2008  Staszewicz | Acute abdominal pain | NA | 88/M | Jejunum (multiple) | NA | CT; multiple small bowel giant diverticula surrounded by inflammatory mesenteric fat | Segmental resection of the 35 cm jejunum, including all diverticula, with primary anastomosis | Discharged POD 12 |
| 2009  Borgaonkar | Suddenabdominal pain associated with vomiting | 3days | 65/M | Jejunum (1) | Yes | Sonography; multiple, thick walled a peristaltic small bowel loops with free fluid in abdomen | Exploratory laparotomy  Segmental resection of the affected jejunum, including all diverticula, with primary anastomosis | Discharged POD 10 |
| 2009  Colvin | Episodic, abdominal pain, bloating, anorexia, vomiting and loose stools | 7days | 87/M | Jejunum (NA) | Yes | CT; multiple dilated loops of small bowel surrounding an area of marked soft tissue stranding with multiple small locules of gas | Conservative | Discharged after 4 days of admission |
| 2010  Sakpal | Abdominal pain left lower quadrant and the periumbilical, fever and bilious vomiting | 3days | 25/F | Jejunum (1) | Yes | CT; a thickened jejunal wall with mild dilatation and an air-fluid-containing structure in the left abdomen | Exploratory laparotomy  Segmental resection of the affected jejunum with primary anastomosis | Discharged POD 7 |
| 2010  Butler | Generalized abdominal pain, with three episodes of vomiting | 1day | 82/F | Jejunum (multiple) | Yes | CT; multiple small bowel diverticula with surrounding pockets of free air | Primary closure of two sites of perforated diverticula | recovered |
| 2013  Akbari | Constipation, anorexia, fever, and left sided abdominal pain | 2days | 74/M | Jejunum (multiple) | Yes | no | Exploratory laparotomy  Segmental resection of the affected 20 cm jejunum | Discharged POD 7 |
| 2014  Kavanagh | Right lower quadrant pain | 2days | 63/M | Jejunum (multiple) | Yes | CT; extraluminal gas | Exploratory laparotomy  Segmental resection of approximately 1 m jejunum including 95% of diverticula | Discharged POD 3 |
| 2014  Baksi | Generalized abdominal pain　and absolute constipation | 1-2days | 55/M | Jejunum (1) | Yes | X-ray; dilated loops of small bowel and free gas under the domes of the diaphragm | Exploratory laparotomy  Segmental resection of the perforated jejunum with 　primary anastomosis | Discharged POD 10 |
| 2014  Levack | Sharp abdominal pain localized just lateral and inferior to umbilicus | 3days | 77/F | NA | No | CT; a focally thickened loop of small bowel with a small collection adjacent to the thickened small bowel | Conservative | Discharged after 5 days of admission |
| 2015  Natarajan | Dragging upper abdominal pain, dyspepsia, abdominal discomfort and vomiting | 7days | 58/M | Jejunum (multiple) | NA | X-ray; free air under the diaphragm  CT and ultrasound; multiple diverticula in the small intestine and air under the diaphragm | Segmental resection of the perforated jejunum with primary anastomosis | Discharged POD 10 |
| 2016  Sehgal | Worsening lower abdominal pain associated with nausea and low-grade pyrexia | 2days | 82/M | Jejunum (multiple) | Yes | CT; a hollow viscus perforation with small amount of intra-abdominal free air and intrapelvic free fluid and inflammatory change in the left paramedian mesentery | Exploratory laparotomy  Segmental resection of the perforated jejunum with a side-to-side anastomosis | Discharged POD 7 |
| 2017  Ejaz | Acute abdominal pain and fever | on the day | 87/M | Jejunum (multiple) | NA | CT; mesenteric fat stranding and a small pocket of extraluminal gas adjacent to a jejunal diverticulum | Conservative | Discharged after 5 days of admission |
| 2017  Karas | Abdominal pain that was periumbilical in origin and migrated to the right lower quadrant | 1day | 69/M | Terminal ileum(multiple including jejunum) | No | CT; thickening of the terminal ileum, caecum and ascending colon and a small foci of extraluminal gas surrounding the terminal ileum | Initially conservative but worsened in the next 24 hours  Exploratory laparoscopy → converted to an open laparotomy  Segmental resection of severely inflamed small bowel with primary anastomosis | Unremarkable |
| 2018  Syllaios | Acute abdominal pain, fever and abdominal distention | 2days | 75/M | Jejunum (multiple) | Yes | CT; a small amount of extraluminal air (2 cm in diameter) adjacent to the jejunum | Segmental resection of the affected 45 cm jejunum with primary anastomosis | Discharged POD 6 |
| 2018  Kagolanu | Acute whole abdominal pain and radiated to the right flank area, nausea, vomiting | 2days | 91/M | Jejunum (multiple) | Yes | CT; small bowel diverticula with inflammation and a contained micro-perforation | Conservative | Treated within 2 days |
| 2018  Alves | Acute diffuse abdominal pain, associated with nausea and vomiting | 1day | 74/F | Jejunum (multiple) | Yes | X-ray; free gas under the right hemidiaphragm and  nonspecific gaseous distension of the small bowel | Exploratory laparotomy  Segmental resection of the affected 20cm jejunum with primary anastomosis | Discharged POD 22 |
| 2019  Jambulingam | Suprapubic pain radiating to the epigastrium, sweats, chills and nausea | on the day | 63/F | Jejunum (2) | No | CT; significant inflammatory infiltrate surrounding large jejunal diverticulum which was localized to the surrounding mesentery | Conservative | Discharged after 2 days of admission |
| 2020  Kunishi | Periumbilical pain and fever | on the day | 40/F | Jejunum (multiple) | No | CT; localized extraluminal air and panniculitis adjacent to the jejunum diverticula | Conservative | Discharged after 6 days of admission |
| 2022  Leigh | Sudden abdominal pain starting in the lower abdomen and nausea | 2days | 59/F | Jejunum (multiple) | No | CT; a jejunal loop with a large diverticulum on the mesenteric side with associated diverticulitis and perforation | Segmental resection of the 27 cm jejunum with primary anastomosis | Discharged POD 6 |
| 2021  Ben | Mild to moderately intense abdominal pain and vomiting | 2days | 52/M | Jejunum (1) | Yes | CT; a jejunal diverticulum with surrounding inflammatory changes in the mesenteric fat | Initially conservative but worsened in the next 72hours  Exploratory laparoscopy  Segmental resection of the 10 cm jejunum with primary anastomosis | NA |
| 2021  Rajaguru | Fever, chills and progressive abdominal distension | 5days | 74/M | Ileum (1) | Yes | CT; inflammatory changes in the right iliac fossa with the presence of extra-luminal gas locules | Exploratory laparoscopy  Laparoscopic assisted right hemicolectomy | Discharged POD 4 |
| 2022  Ponce | Colicky abdominal pain | 7days | 83/M | Jejunum (multiple) | Yes | CT; air-fluid distention of the entire small bowel and small mesenteric collection | Exploratory laparotomy  Segmental resection of the 10 cm jejunum and ostomy | Died 6 hours later |
| 2022  Mejri | Severe, generalized abdominal pain with vomiting | 1day | 60/F | Jejunum (multiple) | Yes | NA | Segmental resection of the affected 40 cm jejunum with primary anastomosis | Discharged POD 6 |
| 2023  Jawed | Constipation, nausea, vomiting and localized pain in the lower abdomen | 3-4days | 75/M | Ileum (3) | Yes | X-ray; dilated bowel loops and free air under the right and left domes of the diaphragm | Exploratory laparotomy  Segmental resection of the ileum and a double barrel ileostomy | Discharged POD 4 |
| 2023  Dar | Sudden epigastrium pain and spread to the whole abdomen, anorexia, nausea, vomiting, and absolute constipation | 2days | 38/F | Jejunum (12) | Yes | X-ray; free air under the diaphragm | Exploratory laparotomy  Segmental resection of the affected jejunum, including all diverticula, with primary anastomosis | Discharged POD 10 |
| 2023  Matsuya | Localized lower abdominal pain associated with vomiting | on the day | 73/M | Jejunum (multiple) | Yes | CT; a thickened partial small-bowel wall, stranding of peri-intestinal fat  Repeat CT: revealed extraintestinal gas around the small intestinal diverticula | Initially conservative but worsened in the next 96 hours Segmental resection of the perforated jejunum with primary anastomosis | Discharged POD 11 |
| 2023  Matsuya | Worsening lower abdominal pain | 2days | 73/F | Jejunum (multiple) | Yes | CT; scattered diverticula in the small intestine to the colon, thickened partial small bowel wall, and extra-intestinal gas around the diverticula | Segmental resection of the perforated jejunum with primary anastomosis | Discharged POD 7 |
